# Challenges and Efficacy of Amikacin Liposome Inhalation in Real-World Refractory Mycobacterium avium Complex Pulmonary Disease

**DOI:** 10.7759/cureus.95192

**Published:** 2025-10-22

**Authors:** Toyoshi Yanagihara, Yusuke Osaki, Takato Ikeda, Akira Nakao, Yuki Shundo, Naoki Hamada, Noriyuki Ebi, Hiroyuki Inoue, Masaki Fujita

**Affiliations:** 1 Department of Respiratory Medicine, Fukuoka University Hospital, Fukuoka, JPN

**Keywords:** alis, amikacin liposome inhalation suspension, fibrocavitary, mycobacterium avium complex, nodular bronchiectatic

## Abstract

Amikacin liposome inhalation suspension (ALIS) has emerged as a therapeutic option for patients with refractory *Mycobacterium avium* complex (MAC) pulmonary disease. This retrospective, observational study presents real-world data on the efficacy and safety of ALIS in 12 patients treated at our institution between July 2021 and November 2024. The cohort consisted predominantly of older patients (median age 72 years) with low BMI (median BMI 18.3). Radiographic patterns included nodular bronchiectatic (NB) and fibrocavitary (FC) types, with 75% of patients exhibiting cavitary lesions. Prior treatment regimens primarily involved azithromycin- or clarithromycin-based therapies, supplemented with sitafloxacin, with a median treatment duration of five years. ALIS therapy was discontinued in two cases due to adverse events, including fever and bronchospasm, while eight patients experienced hoarseness, and two reported hearing impairment or dizziness. Symptomatic improvement was observed in seven patients, and three of the nine patients who continued treatment for at least six months achieved culture conversion, all of whom exhibited the NB radiographic pattern. These findings highlight the challenges of managing refractory MAC pulmonary disease in a real-world setting and emphasize the importance of considering patient characteristics, including radiographic patterns and baseline health, when initiating ALIS therapy. Adverse events were frequent but manageable with appropriate monitoring and adjustments.

## Introduction

Nontuberculous mycobacterial (NTM) pulmonary disease is a chronic and often progressive infection that carries substantial morbidity and mortality. Among NTM species, *Mycobacterium avium* complex (MAC) is the leading cause worldwide and accounts for approximately 90% of cases in Japan [1]. Patients with MAC pulmonary disease (MAC-PD) often experience prolonged treatment courses, frequent relapses, and impaired quality of life. Standard guideline-based therapy (GBT), consisting of a macrolide, ethambutol, and rifamycin, remains the cornerstone of management [2]. However, despite prolonged administration, treatment outcomes are unsatisfactory, with many patients developing refractory disease or macrolide resistance [3]. These limitations highlight the urgent need for new therapeutic strategies.

Amikacin liposome inhalation suspension (ALIS) was developed to enhance pulmonary drug delivery while reducing systemic toxicity compared with intravenous aminoglycosides. The Phase 3 CONVERT trial demonstrated that adding ALIS to GBT improved culture conversion rates in patients with treatment-refractory MAC-PD [4], with consistent findings observed in the Japanese subpopulation [5]. Based on these results, the Infectious Diseases Society of America (IDSA) guidelines recommend ALIS as an add-on therapy for patients with refractory MAC-PD [6]. Nevertheless, treatment discontinuation due to adverse events such as dysphonia, cough, and hypersensitivity pneumonitis has been frequently reported, highlighting the importance of balancing efficacy and tolerability in clinical practice.

Although clinical trials provide robust evidence, clinical trial populations such as those in CONVERT are typically selected under strict inclusion and exclusion criteria, and therefore may not reflect the broader, more heterogeneous patient population encountered in daily clinical practice. In Japan, where MAC-PD prevalence continues to rise, real-world data are crucial to inform patient selection and optimize treatment strategies. Here, we conducted a retrospective study to evaluate the efficacy and safety of ALIS in a cohort of Japanese patients with refractory MAC-PD treated at a tertiary referral center. By analyzing clinical characteristics, radiographic patterns, and microbiological responses, we aimed to identify factors associated with treatment outcomes and provide insights into the practical use of ALIS in routine care.

## Materials and methods

Study design and patients

We conducted a retrospective observational study at Fukuoka University Hospital, Fukuoka, Japan, to evaluate the efficacy and safety of ALIS in patients with refractory MAC-PD. The primary objective was to assess the efficacy of ALIS in achieving sputum culture conversion among patients with refractory MAC-PD. The secondary objectives were to evaluate the safety and tolerability of ALIS, to identify potential clinical or radiographic predictors of treatment response, and to describe the profiles and management of adverse events observed during therapy. Eligible patients were adults who initiated ALIS between July 2021 and November 2024 after failing to achieve sputum culture conversion with at least six months of GBT. Refractory disease was defined according to international guidelines as persistent positive MAC cultures despite appropriate multidrug therapy [6]. Patients were included if they had at least one follow-up sputum culture after ALIS initiation and available clinical data. This study was conducted in accordance with the Declaration of Helsinki and was approved by the Institutional Review Board of Fukuoka University (approval number: U25-02-006).

Sputum collection and culture procedures

Sputum specimens were collected and processed according to the Guidelines for Mycobacterial Examination (The Japanese Society for Tuberculosis and Nontuberculous Mycobacteriosis, 2025) [7].

Pre-Treatment

Each sputum sample was liquefied and homogenized using “Sputazyme” (Kyokuto Pharmaceutical Industrial Co., Ltd., Tokyo, Japan). Decontamination was then performed using the N-acetyl-L-cysteine-sodium hydroxide (NALC-NaOH) method with the MycoPrep mycobacterial processing kit (Becton, Dickinson and Company, Franklin Lakes, New Jersey, United States), followed by centrifugation.

Culture

The processed specimen was inoculated into a liquid mycobacteria growth indicator tube (MGIT) medium (Becton, Dickinson and Company) and monitored for six to eight weeks at 37 °C using the MGIT automated system. Smear microscopy was performed using fluorochrome staining of the concentrated sample, and when positive, solid media were also used for culture. Solid media included Ogawa medium and 7H11-C agar (both from Kyokuto Pharmaceutical Industrial Co., Ltd).

ALIS administration and concomitant medications

ALIS (590 mg/8.4 mL; Arikayce®, Insmed Inc., New Jersey, United States) was administered once daily via the Lamira® nebulizer system (PARI Pharma GmbH, Munich, Germany) in accordance with the manufacturer’s instructions. Patients were instructed and trained in proper inhalation techniques by pharmacists before the start of therapy. All patients received ALIS as an add-on to GBT, which included a macrolide (azithromycin or clarithromycin), ethambutol, and rifamycin. Sitafloxacin was added to the regimen in selected patients with persistent positive cultures.

Data collection

Baseline demographic and clinical data were extracted from medical records, including age, sex, body mass index (BMI), comorbidities, and prior treatment history. Radiographic findings were classified as nodular bronchiectatic (NB) or fibrocavitary (FC) patterns, and the presence of cavitary lesions was recorded. Microbiological data included MAC species, drug susceptibility testing for clarithromycin and amikacin, and serum anti-glycopeptidolipid (GPL)-core IgA antibody levels, when available. Clinical outcomes were assessed by changes in symptoms, sputum culture conversion in patients who continued treatment for at least six months, and radiological findings. Safety outcomes included treatment-emergent adverse events, discontinuation of ALIS, and ototoxic or respiratory complications. Safety was evaluated based on adverse events recorded in medical charts and classified according to the Common Terminology Criteria for Adverse Events (CTCAE) version 5.0 [8].

Handling of missing data

Drug susceptibility results were unavailable for three patients, and GPL antibody levels were not measured in one patient. These missing data were recorded as “not available” and excluded from comparative analyses, without imputation.

Statistical analysis

Continuous variables were summarized as medians with ranges, and categorical variables as frequencies and percentages. Comparisons between patients who achieved culture conversion and those who did not were performed using Fisher’s exact test or Mann-Whitney U test, as appropriate. A p-value <0.05 was considered statistically significant. All analyses were conducted using standard statistical software (GraphPad Prism 10; Dotmatics, Boston, Massachusetts, United States).

## Results

Baseline characteristics of the study subjects

**Figure 1:**
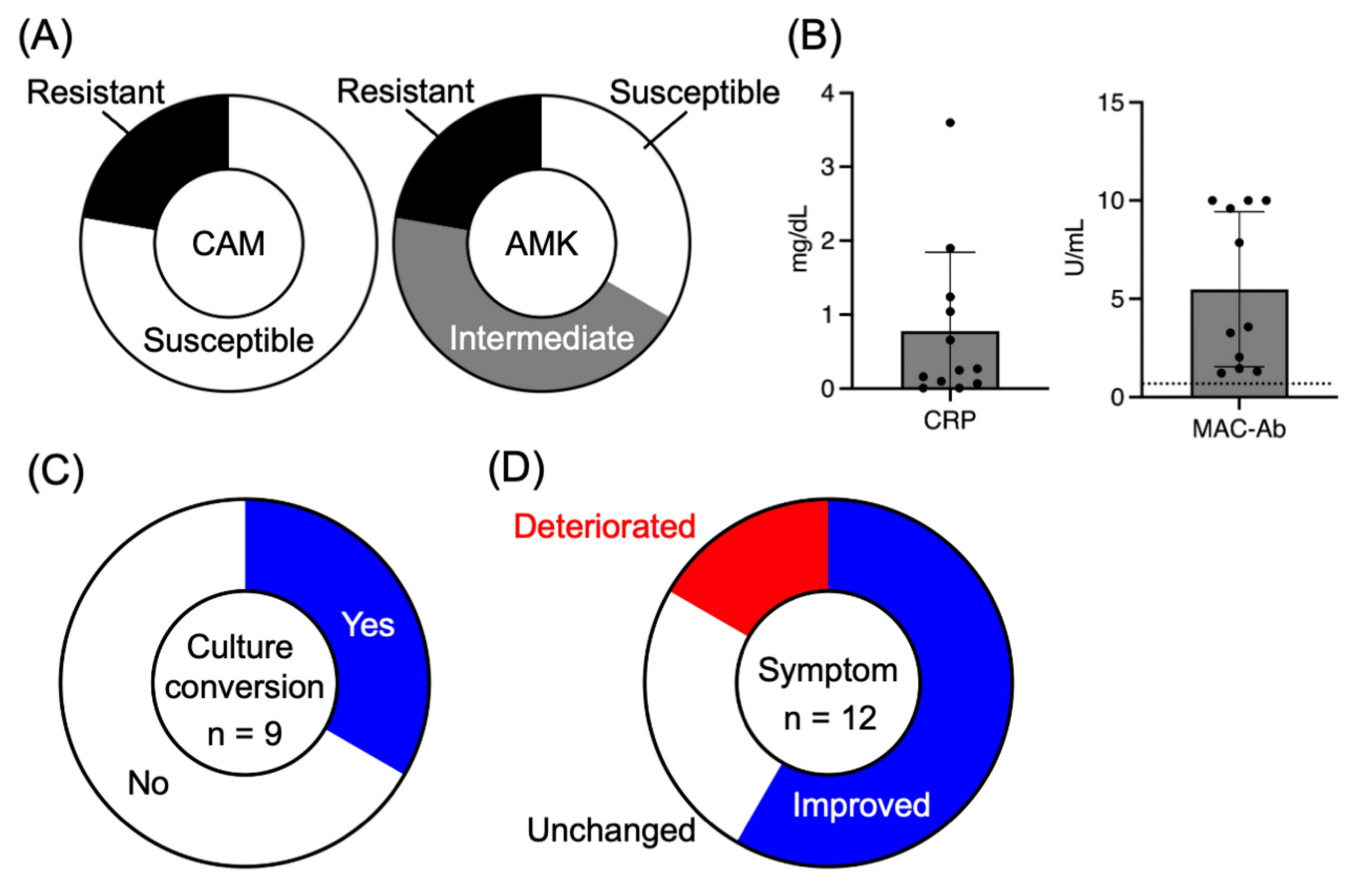
Microbiological and clinical outcomes of patients receiving ALIS. (A) Drug susceptibility profiles of MAC isolates to clarithromycin (CAM) and amikacin (AMK). (B) Baseline serum C-reactive protein (CRP) levels and anti-glycopeptidolipid antibody (MAC-Ab) titers. (C) Proportion of patients who achieved sputum culture conversion within six months of ALIS treatment. (D) Symptomatic changes after ALIS initiation, categorized as improved, unchanged, or deteriorated. ALIS, amikacin liposome inhalation suspension; MAC, *Mycobacterium avium* complex

A total of 12 patients with refractory MAC-PD were treated with ALIS at our institution between July 2021 and November 2024. The median age was 72 years (range, 51-82), and 10 patients (83%) were female. The median BMI was 18.3 kg/m² (range, 15.4-25.1). Infecting organisms included *M. avium* in 10 cases and* Mycobacterium intracellulare *in two cases. The proportions of clarithromycin- and amikacin-resistant strains were both 22% (Figure 1A). Radiographic patterns were divided between FC (n=6) and NB (n=6), and cavitary lesions were observed in nine patients (75%). Most patients (83%, 10 of 12) received clarithromycin-based regimens, while the remaining 17% (2 of 12) received azithromycin-based regimens, often with the addition of sitafloxacin, with a median treatment duration of 5.0 years (range, 1.3-16) before ALIS initiation (Table 1). Baseline serum CRP levels were 0.26 mg/dL (range, 0.01-3.6), and serum anti-GPL-core IgA antibody levels were 3.57 U/mL (range, 1.23-10.0)(Figure 1B). All patients were positive for serum anti-GPL-core IgA antibody (cutoff 0.7 U/mL).

**Table 1:** Clinical profiles and treatment details of patients with MAC pulmonary disease before initiating ALIS therapy (N=12). ALIS, amikacin liposome inhalation suspension; MAC, Mycobacterium avium complex; BMI, body mass index; FC, fibrocavitary; NB, nodular bronchiectatic; CAM, clarithromycin; AMK, amikacin; CRP, C-reactive protein; MAC-Ab, anti-glycopeptidolipid antibody; GBT, guideline-based therapy; AZM, azithromycin; STFX, sitafloxacin

Parameters	Values
Age (years), median (range)	78 (51–82)
Sex (female), n (%)	3 (100)
BMI (kg/m^2^), median (range)	18.8 (18.3-20.1)
Infective MAC isolate, n	
M. avium	10
M. intracellulare	2
Radiographic pattern	
FC type, n	6
NB type, n	6
Cavity, n (%)	9 (75)
Proportion of resistant strains	
CAM, n	33
AMK, n	33
CRP (mg/dL), median (range)	0.26 (0.01-3.6)
MAC-Ab (U/mL), median (range)	3.57 (1.23-10)
GBT, n (%)	
AZM-based	2 (17%)
CAM-based	10 (83%)
STFX-added	7 (58%)
Duration of GBT: (years), median (range)	5.0 (1.3-16)

Treatment outcomes

**Figure 2:**
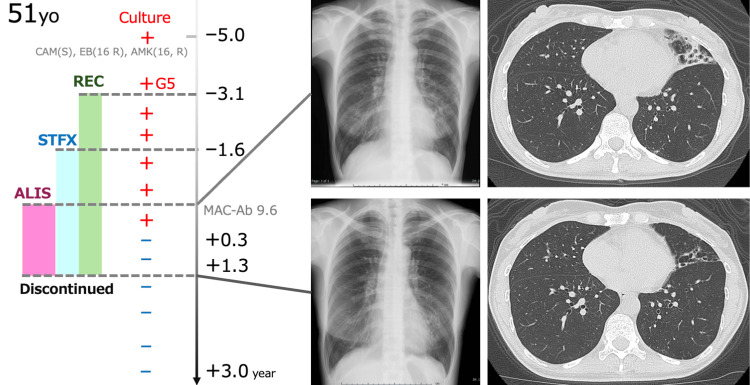
Representative case of culture conversion after ALIS therapy. A 51-year-old female patient with nodular bronchiectatic disease and no cavitary lesions demonstrated radiographic improvement following ALIS initiation. Chest X-ray and CT images before and after therapy illustrate reduced bronchial wall thickening and nodular infiltrates. ALIS, amikacin liposome inhalation suspension

Of the 12 patients, nine continued ALIS for at least six months and were evaluable for microbiological outcomes. The treatment duration ranged from 0.25 to 13 months among the eight patients who completed therapy, while the remaining four patients were still receiving ALIS at the time of analysis (one, nine, 10, 14, respectively). Sputum culture conversion was achieved in three patients (33%) (Figure 1C), all of whom had the NB radiographic pattern (Table 2). In contrast, none of the patients with the FC pattern achieved culture conversion (p=0.0476, Table 2). Symptomatic improvement, including reduced cough and sputum production, was reported in seven patients (58%) (Figure 1D). Representative imaging from a 51-year-old woman with NB-type disease demonstrated radiographic improvement after ALIS therapy (Figure 2).

**Table 2:** Comparison of clinical characteristics between patients with and without culture conversion at six months after ALIS initiation. FC, fibrocavitary; NB, nodular bronchiectatic; CAM, clarithromycin; AMK, amikacin; CRP, C-reactive protein; MAC-Ab, anti-glycopeptidolipid antibody; ALIS, amikacin liposome inhalation suspension

Parameters	Culture conversion	Non conversion	Test static	p-value
Number of patients	3	6		
Age (years), median (range)	78 (51–82)	69 (59–75)	U = 6.0	0.51
Sex: female, n (%)	3 (100)	5 (83)	U = 7.5	0.99
BMI (kg/m2), median (range)	18.8 (18.3-20.1)	18.1 (16.4-23.5)	U = 3.5	0.19
Infective MAC isolate				
M. avium	3	5	OR = not calculable	0.99
M. intracellulare	0	1		
Radiographic pattern				
FC type	0	5	OR = 0.0	0.0476
NB type	3	1		
Cavity, n(%)	1 (33)	5 (83)	U = 4.5	0.46
Proportion of resistant strains				
CAM	33	0	U = 6.0	0.33
AMK	33	25	U = 5.5	0.99
CRP	0.07 (0.01-1.04)	0.75 (0.25-3.6)	U = 3.0	0.16
MAC-Ab	5.82 (2.04-9.6)	2.51 (1.23-10)	U = 5.0	0.78

Safety and tolerability

Adverse events were common but generally manageable. Eight patients (67%) developed dysphonia, which was not formally graded but was considered equivalent to grade 1 in severity, most occurring within the first month of therapy. Two patients (17%) discontinued treatment due to adverse events, both arising within one to two weeks after starting ALIS therapy: one developed fever (grade 2), and the other experienced bronchospasm (grade 2). Symptoms improved after discontinuation of ALIS, and the bronchospasm resolved with short-acting β₂-agonist (SABA) inhalation. Additional events included dizziness (grade 1) (n=2) and hearing impairment (grade 1) (n=2). Laboratory monitoring did not reveal significant nephrotoxicity. Overall, 10 of 12 patients (83%) were able to continue ALIS beyond the initial treatment period with dosing interval adjustment or supportive care.

## Discussion

In this retrospective study, we evaluated the real-world efficacy and safety of ALIS in patients with refractory MAC-PD. Our results showed that the study population was characterized by older age and low body mass index, reflecting real-world clinical practice. Culture conversion was achieved only in patients with the NB pattern, whereas none of the patients with the FC type achieved conversion. This finding is consistent with recent reports from Japan, which have demonstrated that the absence of cavitary lesions is strongly associated with higher conversion rates [9,10]. For example, Urabe et al. reported a 58.3% conversion rate, predominantly in patients without cavities [9]. Kurahara et al. found that conversion occurred in 84% of patients with the noncavitary NB subtype but in only 36% with the cavitary subtype [10]. The higher response rate observed in NB-type disease may be related to its lower bacterial burden and preserved airway structure, which facilitate better delivery and penetration of inhaled amikacin. In contrast, cavitary lesions often harbor dense biofilms and thick caseous walls that impede drug diffusion, potentially reducing ALIS efficacy. Together with our findings, these data suggest that early initiation of ALIS, before disease progression to extensive cavitary involvement, may maximize the likelihood of achieving culture conversion.

Adverse events were common in our cohort, with dysphonia being the most frequent, consistent with prior studies. While this side effect rarely necessitated discontinuation, it often impacted treatment adherence. Several strategies have been proposed for managing ALIS-associated voice disorders. Kurahara et al. recommended morning administration before breakfast to reduce throat burden [11], while other practical measures include gargling with warm water or adjusting inhalation timing [12]. Additionally, alternate-day inhalation has been used in clinical practice to mitigate adverse events [13,14]. These approaches may help patients continue long-term therapy while maintaining quality of life. We previously reported that the incidence of dysphonia was only 20% in another institution, where pharmacists provided structured inhalation guidance to patients [15].

Our findings also highlight broader issues in the management of refractory MAC-PD. Although ALIS significantly increases the chance of culture conversion compared with GBT alone, the overall success rate remains modest, particularly in patients with cavitary disease. Amikacin resistance, often associated with rrs mutations, leads to high-level resistance to amikacin and reduced susceptibility to other aminoglycosides such as kanamycin and tobramycin. These mutations may also coexist with rrl gene alterations linked to macrolide resistance, further limiting available treatment options [16]. Careful patient selection, early intervention before irreversible lung destruction, and close monitoring for resistance are therefore critical.

This study has several limitations. It was a single-center retrospective study with a small sample size, which limits generalizability and precludes multivariate adjustment for potential confounders. The equal distribution between FC and NB patterns, together with the small number of cases, may have limited the statistical power for subgroup comparisons and thus the generalizability of our findings. The results should therefore be interpreted as exploratory and hypothesis-generating.

Taken together, our study adds to the growing body of real-world evidence supporting the use of ALIS in refractory MAC-PD, especially in patients with NB-type disease and without extensive cavities. However, high rates of adverse events and the potential for resistance emergence underscore the need for individualized treatment strategies and further prospective studies in diverse patient populations.

## Conclusions

This retrospective analysis demonstrated that ALIS can be an effective and generally tolerable option for patients with refractory MAC-PD in a real-world setting. The treatment benefit appeared most evident in patients with the NB form of disease, suggesting better response in those with fewer cavitary lesions, though this finding should be interpreted cautiously because of the small sample size. Adverse events such as dysphonia were frequent but generally manageable with appropriate guidance and dose adjustments, while some patients required discontinuation, which could affect adherence. Optimizing inhalation timing, ensuring proper technique, and considering alternate-day dosing may help improve tolerability. These findings support the role of ALIS as an add-on therapy for difficult-to-treat cases and emphasize the importance of early intervention and careful monitoring to achieve better long-term outcomes.
